# Fishery-Induced Changes in the Subtropical Pacific Pelagic Ecosystem Size Structure: Observations and Theory

**DOI:** 10.1371/journal.pone.0062341

**Published:** 2013-04-19

**Authors:** Jeffrey J. Polovina, Phoebe A. Woodworth-Jefcoats

**Affiliations:** Pacific Islands Fisheries Science Center, NOAA Fisheries, Honolulu, Hawaii, United States of America; Hawaii Pacific University, United States of America

## Abstract

We analyzed a 16-year (1996–2011) time series of catch and effort data for 23 species with mean weights ranging from 0.8 kg to 224 kg, recorded by observers in the Hawaii-based deep-set longline fishery. Over this time period, domestic fishing effort, as numbers of hooks set in the core Hawaii-based fishing ground, has increased fourfold. The standardized aggregated annual catch rate for 9 small (<15 kg) species increased about 25% while for 14 large species (>15 kg) it decreased about 50% over the 16-year period. A size-based ecosystem model for the subtropical Pacific captures this pattern well as a response to increased fishing effort. Further, the model projects a decline in the abundance of fishes larger than 15 kg results in an increase in abundance of animals from 0.1 to 15 kg but with minimal subsequent cascade to sizes smaller than 0.1 kg. These results suggest that size-based predation plays a key role in structuring the subtropical ecosystem. These changes in ecosystem size structure show up in the fishery in various ways. The non-commercial species lancetfish (mean weight 7 kg) has now surpassed the target species, bigeye tuna, as the species with the highest annual catch rate. Based on the increase in snake mackerel (mean weight 0.8 kg) and lancetfish catches, the discards in the fishery are estimated to have increased from 30 to 40% of the total catch.

## Introduction

The North Pacific subtropical gyre is a large oceanic gyre bounded on the south by the North Equatorial Current, on the west by the Kuroshio Current, on the north by the Kuroshio Extension Current and the North Pacific Current, and on the east by the California Current [1]. Although low in primary productivity, the warm, vertically stratified oligotrophic waters of the subtropical gyre contain a highly diverse food web populated by tunas, sharks, and billfishes at the top trophic levels [2], [3]. Since the 1950s, large-scale fisheries have targeted the tunas, billfishes, and other large predators in this ecosystem. Several studies have suggested possible ecosystem impacts from fishing [3]–[5]. A comparison of catch, size, and species composition between a research longline survey in the 1950s and observer data from commercial longliners in the 1990s suggested a substantial decline in the abundance of large predators, the mean size of these predators, and some evidence of an increased abundance of formerly rare species [5]. Models of the North Pacific subtropical gyre were generated with Ecopath with Ecosim (EwE) to investigate whether the ecosystem contained any keystone species [3], [4]. The results suggested that there was not any single species group that functioned as a keystone, but that a broad reduction of apex predators as a result of fishing might result in an increase in prey in response to a decreased predation [3], [4]. In effect the fishing fleet is the keystone predator [4]. However, another modeling effort using an EwE model that incorporated some size-class structure found that while fishing decreased predator abundance there was limited evidence of trophic cascades or other ecosystem impacts based on the decline in predators [6].

A more recent analysis of catch rates for the 13 most abundant species caught in the deep-set Hawaii-based longline fishery over the past decade (1996–2006) provided evidence of a top-down response of the North Pacific subtropical ecosystem. Catch rates for apex predators such as blue shark (*Prionace glauca*), bigeye (*Thunnus obesus*) and albacore (*Thunnus alalunga*) tunas, shortbill spearfish (*Tetrapturus angustirostris*), and striped marlin (*Tetrapturus audax*) declined from 3 to 9% per year while catch rates for 4 mid-trophic species, mahimahi (*Coryphaena hippurus*), sickle pomfret (*Taractichthys steindachneri*), escolar (*Lepidocybium flavobrunneum*), and snake mackerel (*Gempylus serpens*), increased by 6 to 18% per year [7].

Ecosystem food webs and models for the central North Pacific subtropical pelagic ecosystem have traditionally been built from a species-specific perspective [2]–[4], [6]. Recent analysis of the temporal ecosystem dynamics used trophic levels derived from species-specific models [7]. However, recent applications of size-based ecosystem models across various ecosystems show they are emerging as a powerful tool, particularly in pelagic environments where predation is more strongly driven by body size than species’ taxonomic identity [8]–[13]. A key advantage to size-based models is that they are based on broad ecological and physiological relationships requiring few region-specific parameter estimates apart from sea surface temperature (SST), the size structure at the base of the food web, and fishing gear selectivity.

In this paper we further examine ecosystem changes in the subtropical pelagic system from a size-based perspective and compare observations from the Hawaii-based longline fishery with simulations from a dynamic size-based ecosystem model.

## Materials and Methods

Catch and fishing effort data from the Hawaii-based longline fishery are collected in two ways. Federally mandated logbooks are required from all fishers licensed in that fishery. The logbooks report daily records of fishing activity including location, catch by species, number of hooks per set, and other data on the fishing operation. While logbook data provides complete coverage from all vessels, it is most reliable for the landed portion of the catch while catches of discarded species including sharks and fishes with low or no economic value are often not recorded. Fishery observers are placed on a subset of all vessels to record all the species caught, fishing effort, and various operational aspects and since 2006 they have recorded the length of every third fish caught. Over the period 1996–2011, 16% of the deep-set trips had observer coverage. However, even with this relatively modest observer coverage, observer and logbook catch rates for the commercial species were highly correlated. For example, over the period 1996–2006, annual catch rates from observer and logbook data had correlations ranging from over 0.93 for albacore, striped marlin, shortbill spearfish bigeye tuna, and pomfret, between 0.80 and 0.89 for mahimahi, ono, and yellofin tuna, 0.78 for skipjack, and 0.76 for blue shark [7].

The Hawaii-based longline fishery consists of two components: the daytime deepset fishery targeting bigeye tuna, and the nighttime shallow-set fishery targeting swordfish (*Xiphias gladius*). The deep-set fishery typically sets hooks between 100 m and 400 m with the median hook depth at about 250 m while the median depth of the deepest hook in the shallow-set fishery is 60 m [14]. Deep sets and shallow sets can be identified based on a very strong bimodal distribution of the number of hooks between floats. Shallow sets use 2–6 hooks per float while deep sets use 20–32 hooks per float [14]. For our analysis we identified deep sets as those with 10 or more hooks per float and shallow sets as those with fewer than 10 hooks per float. The shallow-set fishery occurs primarily in the winter and spring within a narrow band of 28°–32° N latitude. The shallow-set fishery was closed for several years to reduce interactions with sea turtles. This paper focuses exclusively on the deepset fishery occurring throughout the year over a broad geographic region and provides an uninterrupted observed catch and effort time series from 1996. Restricting our analysis to the deep-set fishery provides a relatively standardized depth range and method of gear deployment. This analysis was further restricted to data that were obtained from the core region of the fishing ground defined as bounded by 12°–27° N latitude. In some years, the fishery made excursions as far south as the equator and as far north as 32° N latitude; however, fishing in these areas was inconsistent over the study period.

Over the past two decades fishing effort in the Hawaii-based longline fishing ground and fishing mortality over the basin has increased about fourfold. For example, from 1996 to 2008 the number of hooks set in the Hawaii-based longline fishery increased from 10 million to 40 million. Recent stock assessments for yellowfin and bigeye tuna in the central and western Pacific estimated an increase in fishing mortality from 0.1 in 1990 to 0.3–0.4 in 2010 [15]. Unfortunately, we do not have any estimates of fishing mortality for either target species or the ecosystem that apply specifically to the central North Pacific, the area covered by the Hawaii deepset fishery.

We based this study on the catch and effort data for 23 species defined as those that have a mean catch per unit effort (CPUE) of at least 0.05 fish per 1000 hooks set over the 1996–2011 period. For those 23 species we estimated the species’ mean weights from published length-weight equations by using lengths recorded by observers pooled over the 2006–2011 period (Table S1). Although some species had sufficient lengths to compute annual weights we chose to use mean sizes pooled over the entire time period for all species. The reason was our focus on changes in the ecosystem rather than within-species size structure, and many of our 23 species did not have sufficient length data for finer temporal resolution. An analysis of temporal changes in length for the most abundant tunas and billfishes in the catch is presented in Gilman *et al.* [16].

As a robust indicator of ecosystem size structure, we computed annual combined catch for small species (those with mean weights less than 15 kg) and large species (those with mean weights equal to or greater than 15 kg). The value of 15 kg was determined from the species-specific regressions from Table 1 as discussed in the results section. A generalized additive model (GAM) was fit to these two time series to estimate a standardized CPUE time series for small and large species.

**Table 1:** Change in catch rate estimated from statistically significant (*P*<0.01) linear regressions over 1996–2011, in order by by fish size.

Species	% Annual Change in CPUE^a^ (*P*-value)	Mean Weight in kg (*N*)
Blue Marlin (*Makaira nigricans*)	−5.0 (0.005)	224.0 (1295)
Blue Shark (*Prionace glauca*)	−3.7 (0.004)	106.4 (22856)
Striped Marlin (*Tetrapturus audax*)	−5.0 (0.004)	93.5 (3800)
Shortbill Spearfish (*Tetrapturus angustirostris*)	−4.2 (0.008)	75.7 (4078)
Shortfin Mako Shark (*Isurus oxyrinchus*)	0	48.3 (624)
Swordfish (*Xiphias gladius*)	0	42.0 (1509)
Yellowfin Tuna (*Thunnus albacares*)	0	33.5 (9224)
Opah (*Lampris guttatus*)	−4.1 (0.008)	30.2 (3923)
Bigeye thresher Shark (*Alopias superciliosus*)	0	24.0 (1922)
Unidentified Tuna	0	24.0 (49)
Bigeye Tuna (*Thunnus obesus*)	−2.1 (0.005)	22.5 (41456)
Oceanic White-tip Shark (*Carcharinus longimanus*)	−6.9 (<0.0001)	19.0 (277)
Albacore Tuna (*Thunnus alalunga*)	−6.9 (<0.0001)	17.1 (4718)
Wahoo (*Acanthocybium solandri*)	0	16.4 (4172)
Escolar (*Lepidocybium flavobrunneum*)	12.1 (<0.0001)	12.1 (9817)
Mola (*Ranzania laevis* and *Mola mola*)	0	8.8 (521)
Skipjack Tuna (*Katsuwonus pelamis*)	0	7.9 (9352)
Mahi Mahi (*Coryphaena hippurus*)	0	7.4 (19346)
Lancetfish (*Alepisaurus ferox*)	2.2 (0.026)	7.1 (34186)
Great Barracuda (*Sphyraena jello*)	0	5.9 (1198)
Pomfrets (*Taractichthys steindachneri* and *Brama japonica*)	0	4.9 (14898)
Pelagic Stingray (*Pteroplatytrygon violacea*)	−5.4 (<0.0001)	3.0 (4165)
Snake Mackerel (*Gempylus serpens*)	15.1 (<0.0001)	0.8 (15371)

From left, columns indicate species, annual percent change in CPUE based on linear regression (*P*-values for significant trends in parentheses, insignificant fits denoted by a 0% change), mean species weight as determined from length-weight conversion (number of fish used in length-weight conversion indicated in parentheses).

a from linear fit.

Static size-based models, based on metabolic theory and empirical relationships between body size and trophic level, have been applied to investigate unexploited production and biomass of larger marine animals in the global oceans based on current environmental conditions [17]. Dynamic size-spectrum models can extend this approach by considering the time-dependent and continuous growth and mortality processes that result from size-structured feeding, representative of pelagic ecosystems [8], [12]. They can be used to predict the consequences of fishing mortality and changes in primary production as well as temperature effects on dynamic changes in the community size spectrum [13]. A key attribute of these models is that the probability of a predator of size *M* eating an encountered prey of smaller size *m* is given by a lognormal probability density function, with a mean value representing the preferred predator–prey mass ratio and a standard deviation that represents the breadth of the relative prey mass. A key strength of this approach is that realized predator–prey mass ratios in fish communities do not appear to vary systematically with temperature or primary production in the world’s oceans [18].

A size-based ecosystem model based on the pelagic component of the model detailed in Blanchard *et al.* [19] and adapted for the subtropical pelagic ecosystem [20] was used to simulate the response of the size structure to fishing pressure. Input for the model consists of small (<5 µm) and large (>5 µm) phytoplankton densities, SST, size of entry to the fishery, and gear selectivity as a function of size. We used phytoplankton densities and SST output by the NOAA Geophysical Fluid Dynamics Laboratory prototype Earth System Model 2.1 [20], [21], averaged both spatially (12°–27° N, 180°–140°W) and temporally (1996–2011) so the only variable input to the size-based model is fishing mortality.

## Results

Linear regressions fit to the annual CPUE time series for each of the 23 species found 12 species had statistically significant positive or negative slopes (Table 1). Eight species 16.4 kg or larger had declining CPUE trends while the remaining 6 species 16.4 kg or larger had no significant trend (Table 1). Three species 12.1 kg or smaller had increasing CPUE trends while one, pelagic stingray, had a declining trend and the remaining 5 had no trend (Table 1). The declining species included billfishes, sharks, and tunas, with linear CPUE trend declines ranging from 2 to 7% annually over the 16-year time period. The species with increasing linear CPUE trends were escolar and two noncommercial species, snake mackerel and lancetfish, with increases of about 12, 15, and 2% annually, respectively (Table 1).

**Figure 1:**
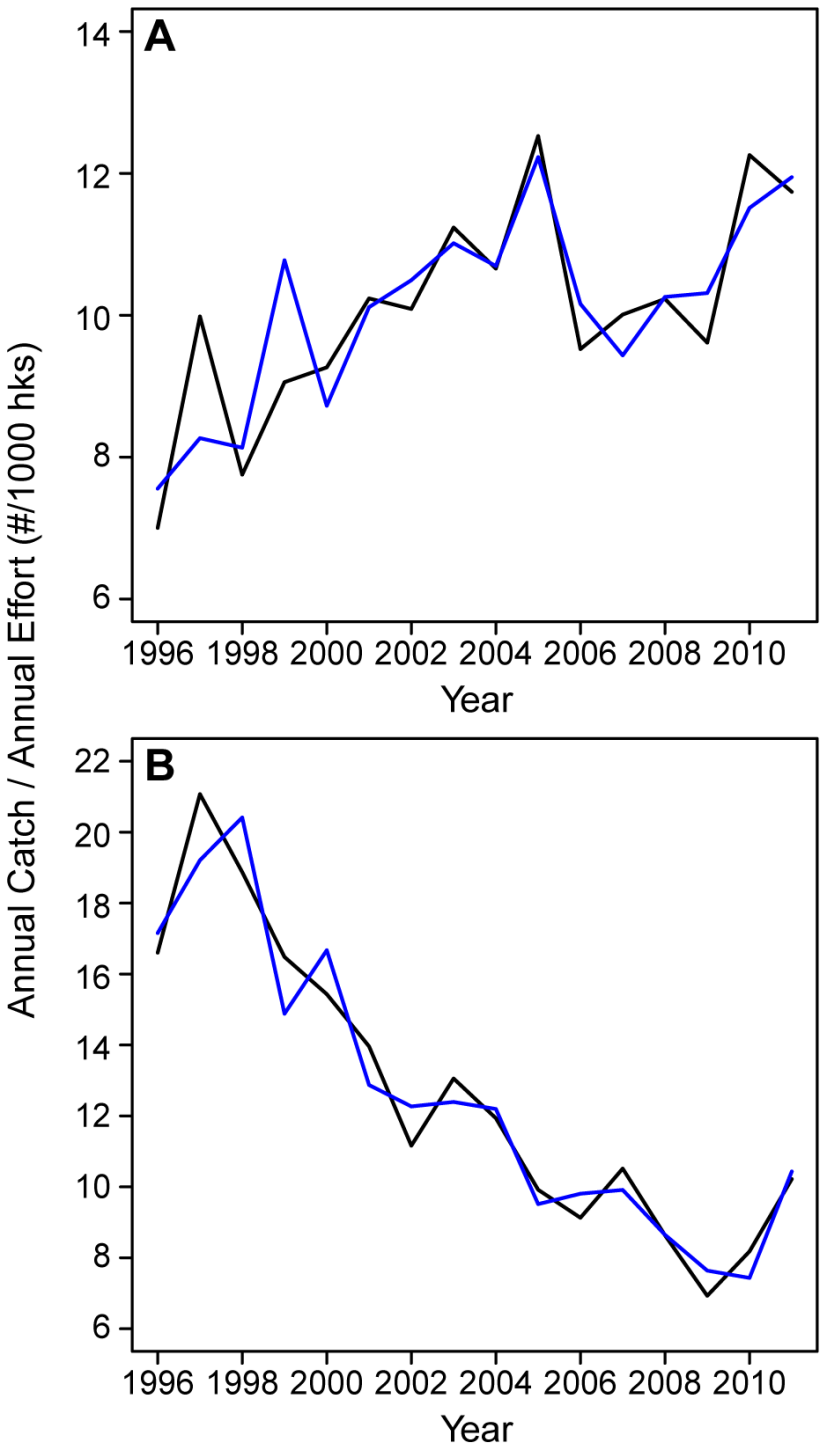
The annual observer and generalized additive model CPUE (# fish per 1000 hooks). Panels indicate (**A**) fishes <15 kg and (**B**) fishes ≥15 kg. In both panels black line represents CPUE from observer data, blue line represents CPUE estimated from the generalized additive model.

**Figure 2:**
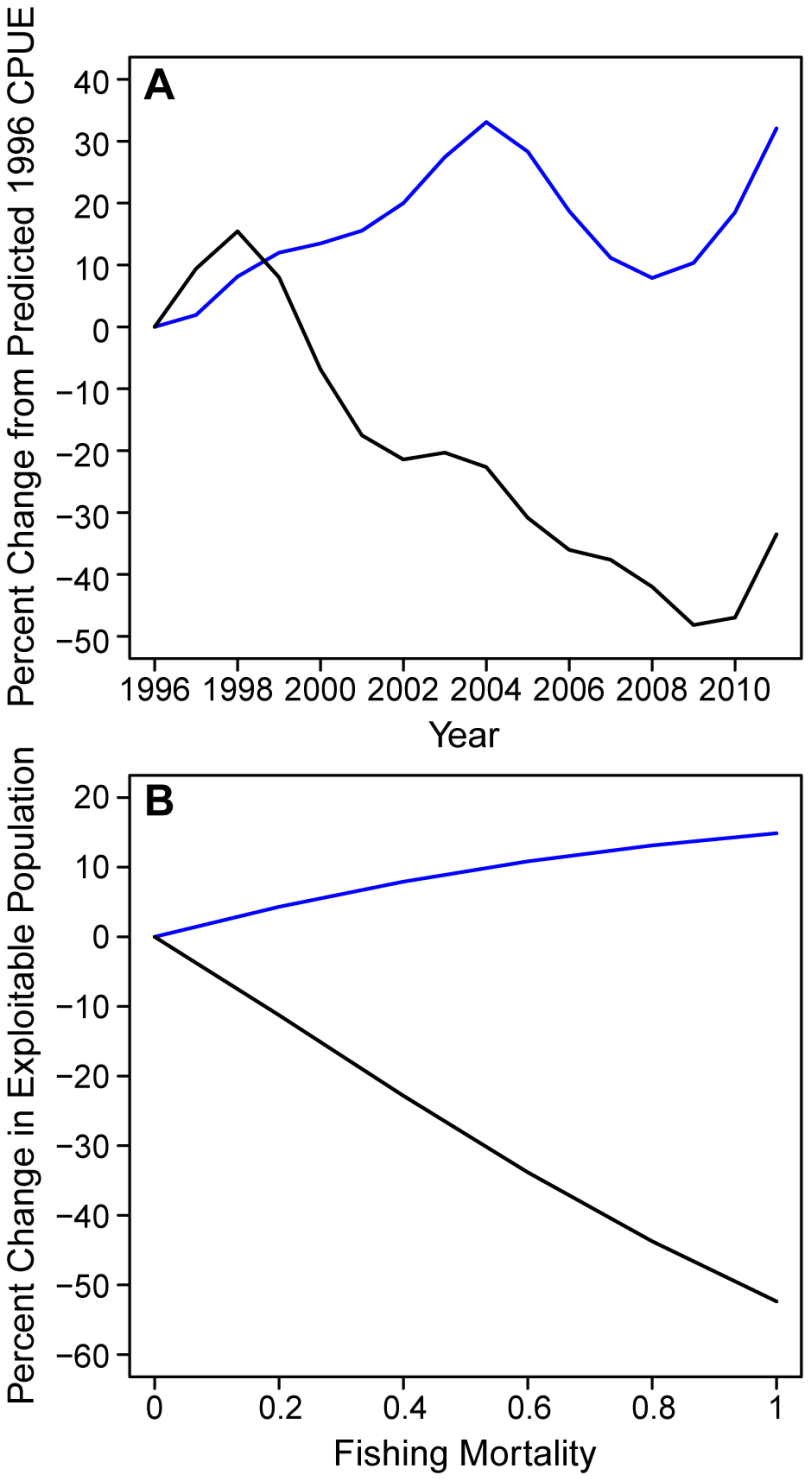
Percent change in standardized CPUE and population size for small and large fishes. Panels indicate (**A**) change in generalized additive model (GAM) standardized CPUE and (**B**) change in size-based model estimated population size for fishes <15 kg (blue) and fishes ≥15 kg (black).

To more rigorously and robustly examine the different CPUE time trends for large and small fishes, we split the catch data into 2 groups consisting of 9 species with mean weights <15 kg, termed small fishes and 14 species with mean weights ≥15 kg, termed large fishes (Table 1). The value of 15 kg used to classify large and small fishes was based on the individual CPUE trends in Table 1 showing that 8 of the 9 species with declining CPUE trends had mean weights of 16.4 kg or larger and all of the species with declining CPUE trends had mean weights 12.1 kg or smaller. A GAM was fit to the catch in numbers per longline set for each size group using independent variables: hooks per set, set latitude, set longitude, SST at set location recorded by the observers, and year (all were significant with *p*<2×10^−16^). The GAM was then used to estimate annual CPUE for each size group by first using the model to estimate catch for each set, then cumulating the estimated catch for each year, and dividing annual estimated catch by annual observed effort to obtain annual estimated CPUE. The resultant estimated annual CPUE time series fits the observed annual CPUE time series quite well with correlations between the observed and estimated CPUEs of 0. 97 for large fishes and 0.86 for small fishes (Fig. 1). From the GAM, standardized annual CPUE time series for large and small fishes are computed as a function of year by replacing the set SST, latitude, and longitude by mean SST, longitude, and latitude from the 16 year period in the GAM and following the steps outlined previously to obtain annual CPUE. This standardized CPUE time series, computed with mean SST, latitude and longitude, is standardized to eliminate any trends in CPUE due to changes in these variables over the 16-year time period. The temporal trend in standardized annual CPUE for small fishes increased about 25% while it decreased by about 50% for large fishes over the time period (Fig. 2a). To check how robust the observer CPUE pattern was we computed standardized CPUEs for large and small fishes from the logbook data. The results, presented as supplemental material (Table S2 and Fig. S1) show the same pattern of about a 50% decrease in large fish CPUE and about a 33% increase in small fish CPUE (Fig. S1) very similar to that seen with the observer data, although the logbook data reports only the commercially valuable species omitting lancetfish and snake mackerel in the small size group and sharks in the large size groups.

**Figure 3:**
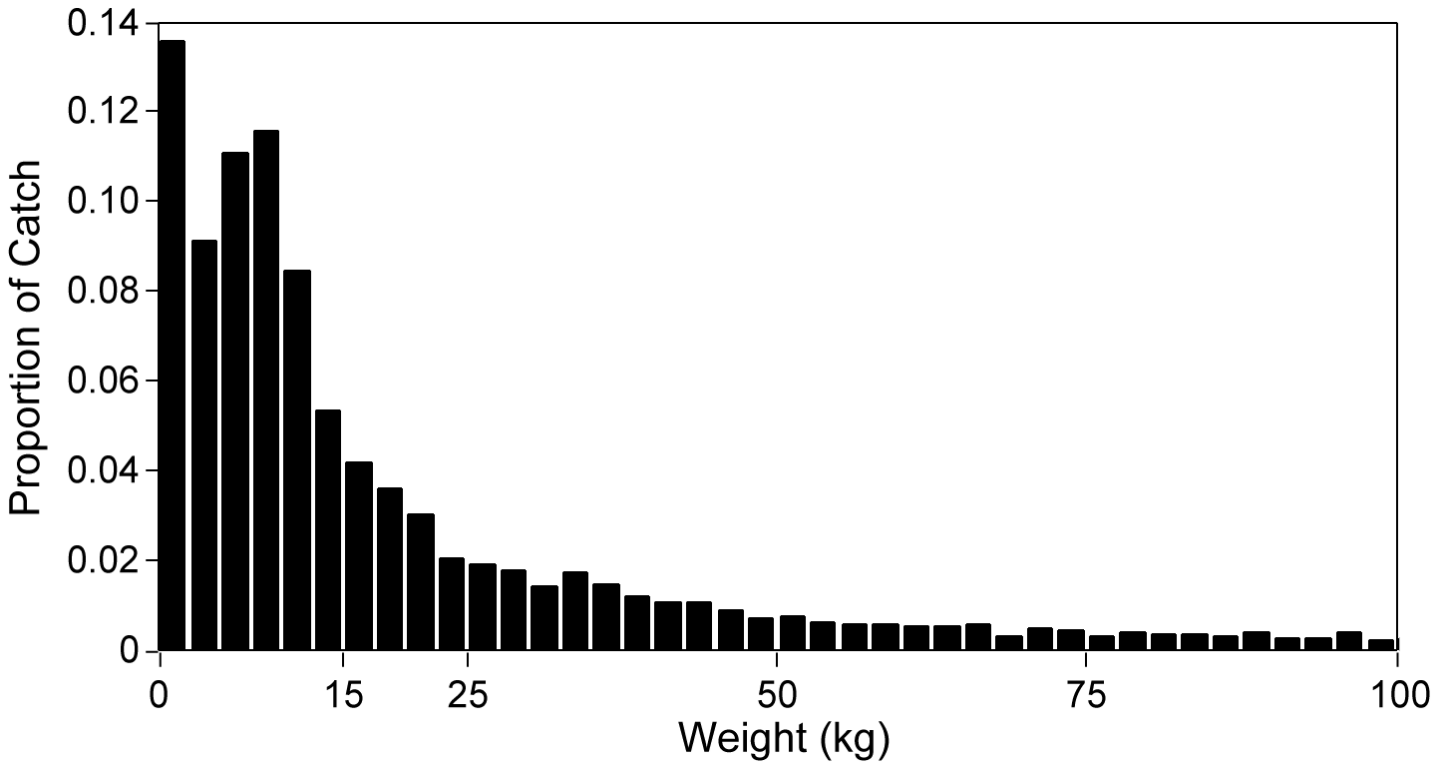
Longline catch weight-frequency distribution. The estimated longline catch weight-frequency distribution for the 23 species listed in Table 1 from the observer data 1996–2011. Distribution truncated above 100 kg.

The mean weights of fishes caught in the Hawaii-based deep-set fishery range from 0.8 kg for snake mackerel to 224 kg for blue marlin (Table 1). Thus for the size at entry to the fishery we use 1.0 kg. To estimate the selectivity of the gear we examine the weight-frequency distribution. The weight-frequency distribution of the catch pooled over the 16-year period shows a typical exponential frequency decline with weight above about 15 kg suggesting that fish above this size are largely fully exploited while this is not the case for smaller fishes (Fig. 3). To further define a selectivity function our pelagic size-based model was run with fishing mortality (F) for F = 0.4 and F = 0.6 to generate catch size distributions. We found a simple size selectivity function where fishes greater than 15 kg experience the full level of F while for fishes in the range of 1–15 kg, F is one fourth the level for the larger fishes generated catch size structures similar to that in Fig. 3.

Using this size selectivity function we compute population abundance as a function of F with the size-based model. As F increases from 0 to 1.0, the population of large fishes declines about 60%, relative to F = 0, while the population of small fishes increases about 20%, relative to F = 0 (Fig. 2b). The standardized fishery CPUE derived from the GAM shows the same pattern of a decline in large fish CPUE and concurrent increase in small fish CPUE (Fig. 2a). Further, both observed and model trends show the decline in large fishes is substantially greater than the increase in small fishes (Fig. 2).

**Figure 4:**
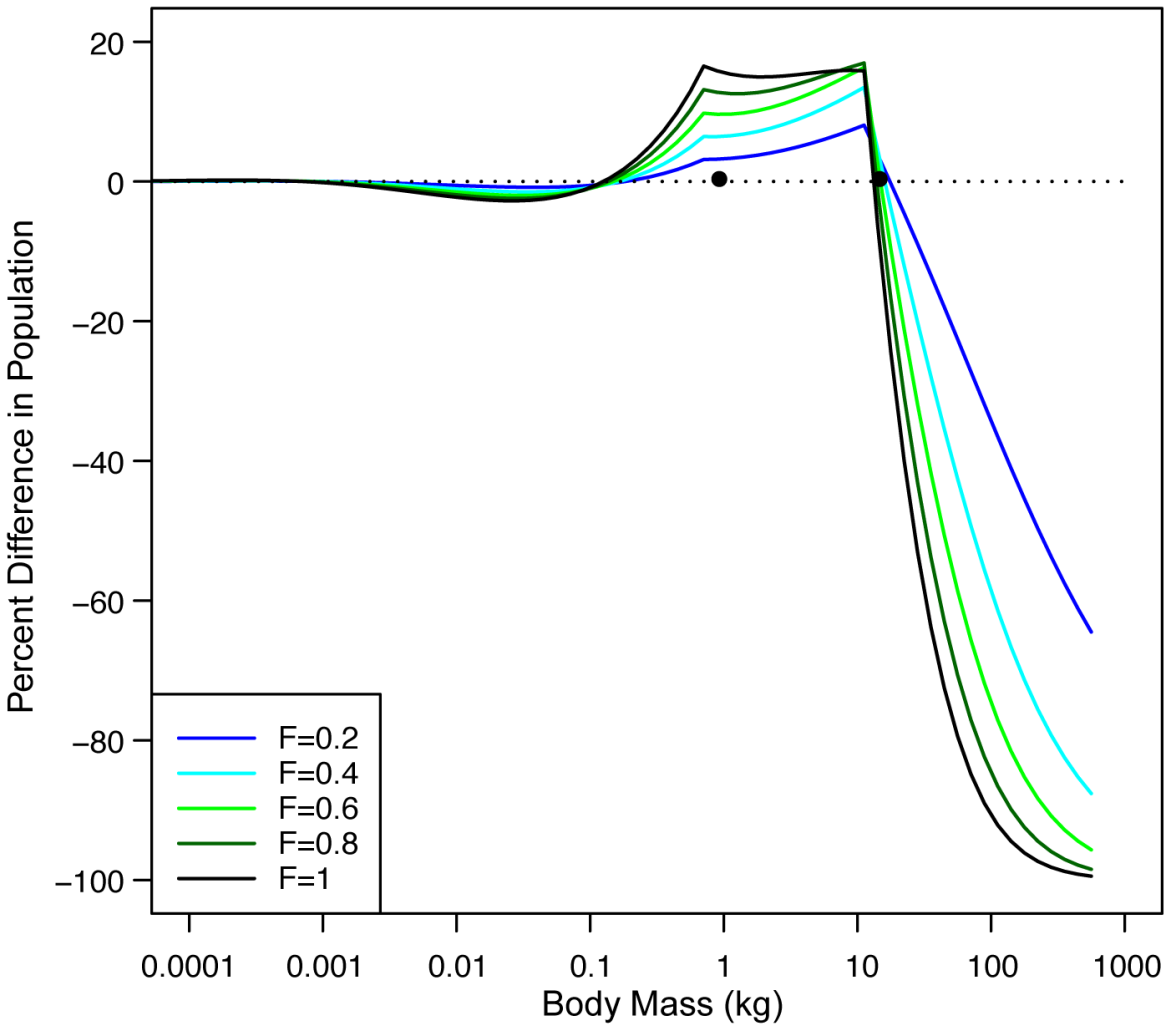
Change in fished ecosystem size structure relative to unfished ecosystem size structure. The percent change in ecosystem abundance by size between the unfished size structure and the fished size structure for F ranging from 0.2 to 1.0. The dots are located at 1 and 15 kg to indicate the size at entry to the fishery and the size of full recruitment.

Next we use the size-based model to examine the change in the entire ecosystem (fished and unfished) size structure in response to various fishing levels. We express the change in ecosystem size structure as a percent change in the size frequency distribution in the absence of fishing relative to that of a fished ecosystem for various levels of F. The change in ecosystem size structure as a function of F shows a decline in the abundance of fishes larger than15 kg, the size of full recruitment to the fishery, and an increase in abundance of fishes within the size range 0.1–15 kg (Fig. 4). For any level of F, the magnitude of the increase in the 0.1–15 kg size range is less than the decline above 15 kg (Fig. 4). A portion of the small size class (1–15 kg) is also fished so this group is responding to both fishing and top-down impacts. For organisms weighing less than 0.1 kg, there is a very slight decrease in abundance but essentially the top-down or size-based cascade has only one cascade with declines for fishes greater than 15 kg resulting in increases for fishes between 0.1–15 kg (Fig. 4).

**Figure 5:**
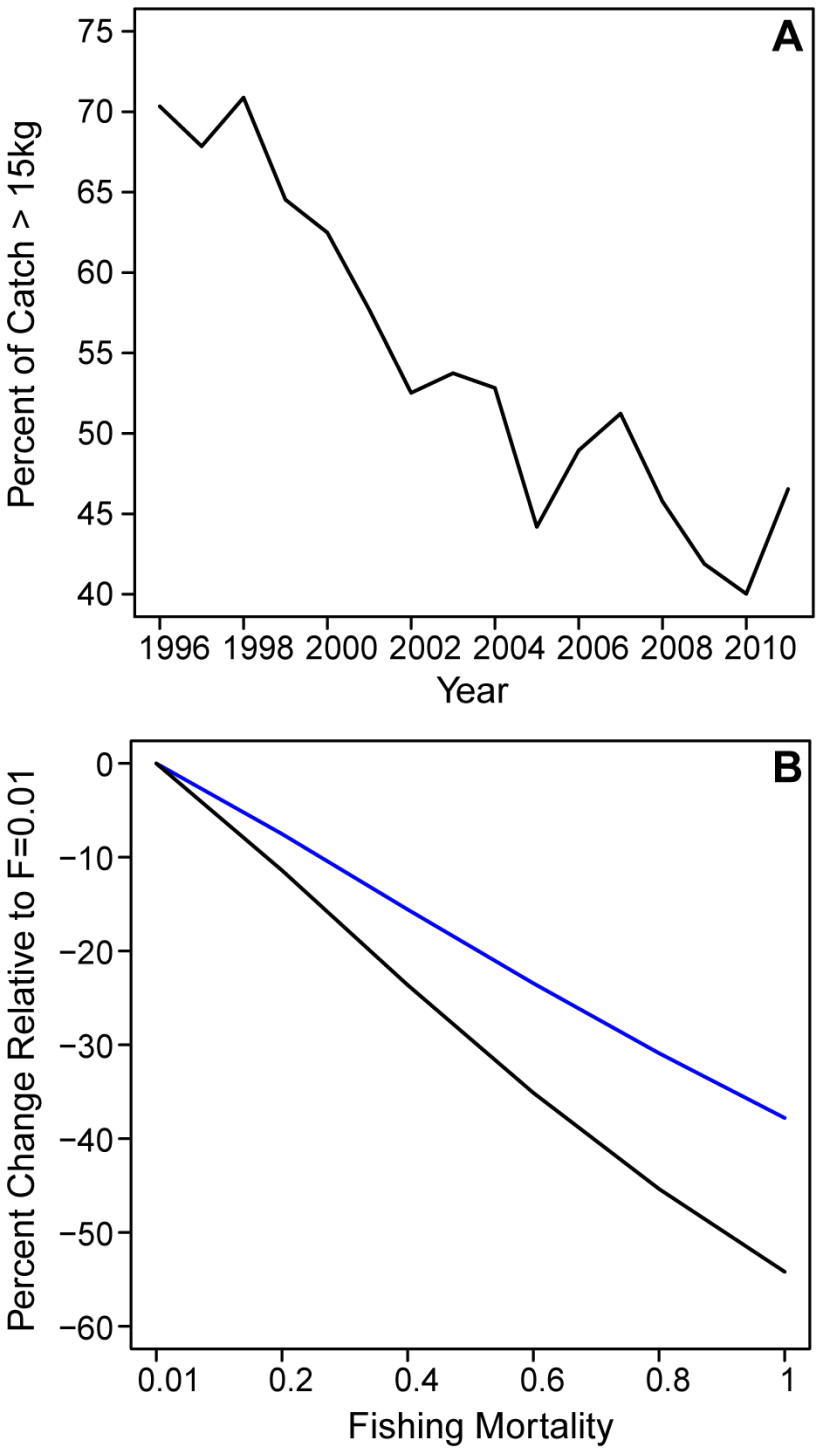
Percent change in catch and biomass. Panels indicate (**A**) the percent of the observed catch >15 kg, by year and (**B**) the percent change in the size-based model large fish catch and biomass relative to F = 0.01 for F ranging from 0.2 to 1.0 for the proportion of catch ≥15 kg (blue) and the proportion of exploitable biomass >0.1 kg that is ≥15 kg (black).

Size-based indicators derived from catch data including the mean size of the catch or the proportion of large fishes have been proposed as useful indicators to monitor fishery trends and ecosystem impacts. In the presence of a size-based cascade, indicators based only on catch data will necessarily underestimate the impact from fishing on the ecosystem size structure. For example, from the observer data we can monitor the change in the size structure of the catch as the proportion of the catch greater than 15 kg. In 1996 about 70% of the catch was greater than 15 kg but this proportion has declined over time to about 45% by 2011, roughly a 25% decline (Fig. 5a). The size-based model predicts that as F increases from 0.01 to 1.0 the proportion of fishes larger than 15 kg in the catch will decline about 30% (Fig. 5b). This is similar to the decline seen in the observer data. However, we have seen from the size-based model that fishing impacts the ecosystem size structure down to 0.1 kg. Thus a more complete measure of the change in the size structure due to fishing would be to compute the ratio of the population of fish larger than 15 kg relative to all fish larger than 0.1 kg. While we can’t do this with catch data, we can with the size-based model. This ratio declines, as F goes from 0.01 to 1.0, by about 55% or almost twice the decline seen in the model catch data since not only does the proportion of large fish decrease but smaller ones increase (Fig. 5b). Thus the change in ecosystem size structure due to fishing may be underestimated if computed from fisheries catch data alone.

**Figure 6:**
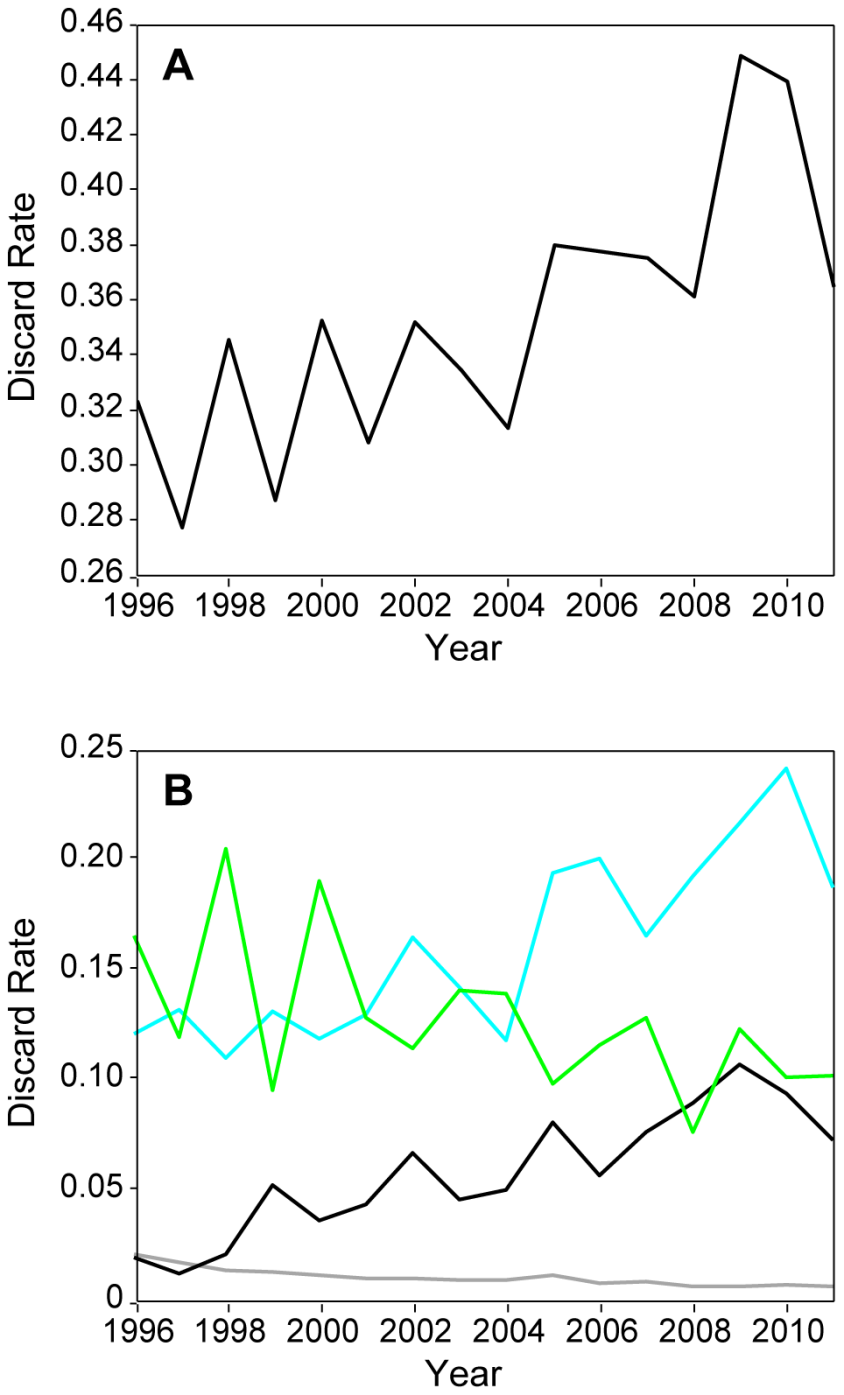
The proportion of the total observed catch estimated to be discarded. Panels indicate (**A**) the combined ratio of estimated discards consisting of the catches of pelagic stingray, snake mackerel, lancetfish, and 95% of the shark catch to total catch and (**B**) the ratio by species of Lancetfish (blue), snake mackerel (black), pelagic stingray (grey), and 95% of the shark catch (green) to total catch.

The rise in catch rates of noncommercial snake mackerel and lancetfish has an impact on the discards in the fishery. The major components of discards in the fishery are the two non-commercial fishes, lancetfish and snake mackerel, which are 100% discarded, and sharks of which about 95% are discarded based on logbook records. Pelagic stingrays are also discarded but their contribution to the total discards is minimal (Fig. 6). Using these discard proportions and the catch for these 3 species from the observer data, we estimate that in 1996 about 30% of the total catch was discarded while by 2011 this proportion had increased to nearly 40% with about one third of the total catch consisting of snake mackerel and lancetfish (Fig. 6).

## Discussion

Evidence of fishing impacts to marine ecosystems, especially reductions in trophic structure (fishing down the food web), has been widely reported [22]. Further, decreases in the catch rates of large fishes and increases in the catch rates of small fishes based on longline catches in the central North Pacific between 1950s and 1990s has been previously reported [5]. Our results indicate this trend has continued through 2011. Further, we show that the temporal change in the species and size composition seen over the past 16 years in the Hawaii-based longline observer data is consistent with and can be explained by a size-based model. This suggests that size-based predation is the dominant mechanism in structuring the subtropical pelagic ecosystem, at least the upper trophic levels caught in the deep-set fishery. Earlier work [7] used a species-based model (EwE) to explain this temporal trend as a top-down response using estimated trophic level instead of size. Our current results and the previous ones are consistent in describing the ecosystem change as a top-down response. The fact that two different model approaches reach the same conclusion is seen as positive. We see the current results as a step forward in that a conceptually (size-based predation) and operationally (requiring many fewer estimated input parameters) simpler model explains the observed ecosystem changes.

One implication of this result is that we have a model-based description of the impact of fishing on the entire ecosystem size structure. The model describes a one-step size-based cascade where a reduction of fishes above the size that is fully exploited by the fishery increases the abundance of organisms from about the size of full entry to the fishery down to about 2 orders of magnitude in size but results in little impact on smaller micronekton and plankton. The reason the top-down impact reaches sizes as small as 0.1 kg is predators have a mean prey size that is 1/100^th^ their weight with a lognormal distribution around that mean. The prey of fishes above 15 kg would have a mean size 0.15 kg and larger. The reason the size-based cascade, expressed as a percent of ecosystem abundance, diminishes with declining size is the ecosystem abundance increases exponentially as size decreases. Thus predation impacts from a fixed larger size represent a smaller and smaller fraction of the prey population as size decreases.

A key result of this work is the observation that the impacts to size structure extends to sizes below those caught in the fishery, and hence catch-based indicators will underestimate the impact of fishing on the ecosystem size structure. Further, unless there is a targeted sampling for the sizes below the size at entry to the fishery these changes will not be recognized. Size-based models can help to more fully represent the full ecosystem impact of fishing on size structure.

One exception to the pattern of a decline in CPUE of large fishes and concurrent increase for small fishes is the pelagic stingray (mean weight 3 kg) that exhibited a 5.4% annual decline. However, an earlier study [5] found that this species increased in the central Pacific longline catches between 1950s and 1990s and attributed this change to a reduction in predation due to declining shark abundance. Fishing effort since the 1990s has continued to increase and while the rays are not retained they are often severely damaged in the release process [23]. Thus their increased bycatch mortality may exceed the decreased predation mortality resulting in a population decline. Pelagic stingrays have been characterized as one of two elasmobranchs with the lowest risk of extinction due to their resilient life history characteristics [23] but our data suggest this may not be the case.

A recent analysis of temporal trends in catch rates of tunas, sharks, and billfishes based on a GAM using observer data the Hawaii longline fishery documented strong general declining trends in standardized catch rates for bigeye, yellowfin and albacore tunas, blue and oceanic white tip sharks, shortbill spearfish and striped marlin [16]. Our results for these species, just based on annual CPUEs but from a more geographically restricted region and over a slightly different time period, also showed significant declining trends in these species with the exception of yellowfin tuna where our estimated linear trend was not statistically significant. Additionally, Gilman *et al*. [16] looked at trends in length for tunas and billfishes and found the lengths significantly increased over time due to the distributions of length classes having shifted towards larger fish. The authors suggest reasons for this shift may include operational changes in the fishery and/or increased catches of juveniles in the purse seine fishery [16]. Initially, this shift to larger fish seems contrary to our finding of an ecosystem shift to small-sized fishes but the difference is a within-species vs. between-species comparison. We used mean weights averaged over the entire time series for each of the 23 species and described the shift in the ecosystem size structure as the shift in the relative abundance of small and large species. We did not examine temporal size trends within species, as many of our 23 species did not have sufficient length data. However, looking at the modest within species length changes presented in Gilman *et al*. [16] relative to the pretty substantial changes in the proportions of large and small fishes in the catch data we conclude that the main change to ecosystem size structure comes from changes in relative abundance between large and small species and not the smaller changes in size within species.

An ecosystem approach to fisheries management looks at fishery impacts to the entire ecosystem. Clearly the longline fishery is changing the subtropical ecosystem size structure. Time series of CPUEs computed separately for the pooled small and large fishes represent an informative ecosystem indicator of this trend and should be monitored and reported in any analysis of the fishery. Current reporting in the fishery shows only catch and catch rates in numbers of fishes so managers are not as likely to be aware of the greater decline in weight per effort compared to numbers per effort, and the former may be more closely related to economics of the fishery. Lastly this work shows the value of observer data, which unlike the more commonly collected logbook data, provides information on bycatch and discarded species that contributes to a more complete understanding of ecosystem dynamics.

Our size-based model does not suggest any obvious threshold in changes to an ecosystem size structure that could serve as a management target. A recent meta-analysis of global fisheries explores tradeoffs between multispecies maximum sustainable yield (MMSY) and the collapse of individual stocks [24]. Their model finds that for a wide range of exploitation rates ranging from 0.25 to 0.60 the resultant catches equal or exceed 90% of the MMSY, but with an exploitation rate of 0.60 almost half the species in the ecosystem are expected to collapse, while with an exploitation rate of 0.25 less than 10% of the species are expected to collapse [24]. Thus in a multispecies context, taking into consideration aspects of ecosystem structure and function, the exploitation rate that achieves maximum sustainable yield should be considered an upper limit rather than a management target [24]. Unfortunately our observer data represents only a small portion of the Pacific pelagic fishery so estimating MMSY and the corresponding F is problematic. This analysis needs to be conducted on the basin-scale by the appropriate regional fisheries management organizations. The sharp decline of stingrays and oceanic white-tip shark presents concern of collapse for these species. Currently management of the longline fishery is based on single species basin-wide quotas for yellowfin tuna, bigeye tuna, and striped marlin set by the Western and Central Pacific Fisheries Commission. To the extent that these quotas cap fishing effort and mortality on all species they could prevent further ecosystems impacts. Further, ways to reduce the estimated 40% discard rate in the fishery should be a management focus as well. Lastly, this work highlights the critical importance of observer data in monitoring ecosystem changes.

While this paper has focused on changes in ecosystem structure it is clear that with increases in escolar and snake mackerel CPUEs of 12 and 15% per year respectively and declines in pelagic stingray and oceanic white-tip CPUEs of 5.4 and 6.9% per year respectively we are also seeing changes in the ecosystem composition with potential significant impacts on ecosystem function.

Lastly, while we have seen evidence of changes at the base of the ecosystem in the subtropical Pacific over the past decade, they have been modest relative to the substantial increase in fishing effort [25], [26]. However, going forward the impact of climate change has been projected to increase its ecosystem impact and shift the subtropical ecosystem size structure toward smaller sizes even if fishing effort remains constant [20], [27]. Thus the combined impacts of increased fishing effort and future climate change are projected to be additive and accelerate a shift of ecosystem size structure to smaller sizes. The time series of CPUE for our small fishes group shows considerably more interannual variation than the large fishes group. Many of these small fishes have faster growth rates and shorter life spans than the larger fishes and hence may be more responsive to interannual environmental changes. Thus a shift to smaller fishes may result in greater interannual variation in the longline fishery CPUE.

## Supporting Information

Figure S1 **The annual logbook and generalized additive model CPUE (# fish per 1000 hooks).** Panels indicate (**A**) fishes <15 kg and (**B**) fishes ≥15 kg. In both panels black line represents CPUE from logbook data, blue line represents CPUE estimated from the generalized additive model.
(TIF)

Table S1 Mean species weight, length, and length-weight conversion factors. From left, columns indicate species, mean species weight as determined form length-weight conversions, mean length from those recorded by observers measuring every third fish from 2006–2011, *a*, *b*, and reference listing length-weight conversion factors. To convert length (*L*) in cm to weight (*W*) in g, the equation *aL^b^ = W* was used.
(DOCX)

Table S2 Change in logbook catch rate estimate from statistically significant (*P*<0.01) linear regressions over 1996–2011, ordered by fish size. From left, columns indicate species, annual percent change in CPUE based on linear regression (*P*-values for significant trends in parentheses, insignificant fits denoted by a 0% change), and mean species weight as determined form length-weight conversion.
(DOCX)
