# Supplementary figures and images for: Fishery-Induced Changes in the Subtropical Pacific Pelagic Ecosystem Size Structure: Observations and Theory
Source: PLoS One. 2013 Apr 19;8(4):e62341. doi: 10.1371/journal.pone.0062341 (PMC3631147; doi:10.1371/journal.pone.0062341)

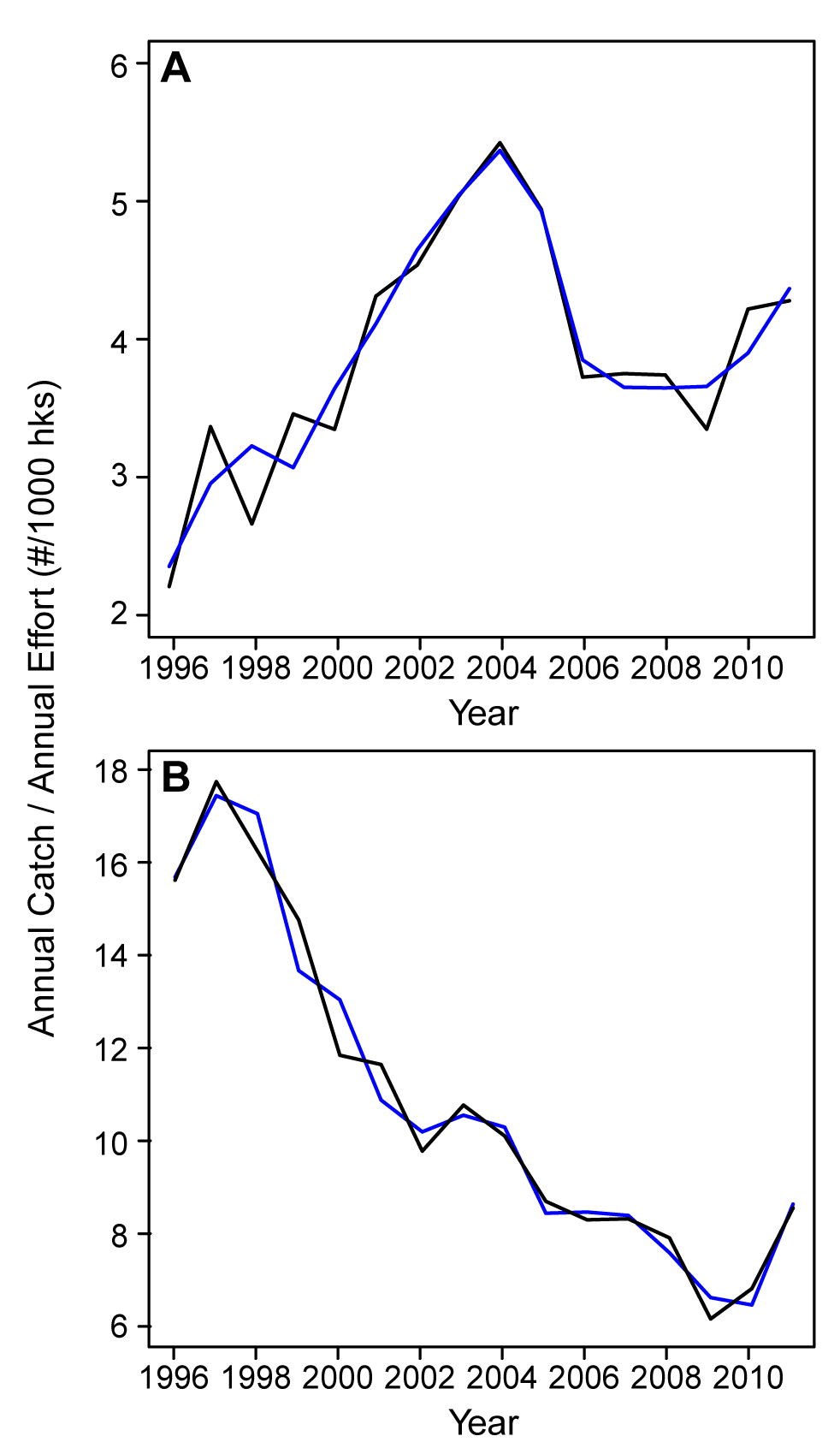

Supplement: Figure S1 — The annual logbook and generalized additive model CPUE (# fish per 1000 hooks). Panels indicate (A) fishes <15 kg and (B) fishes ≥15 kg. In both panels black line represents CPUE from logbook data, blue line represents CPUE estimated from the generalized additive model. (TIF) [file pone.0062341.s001.tif]
