# Supplementary material for: Fishery-Induced Changes in the Subtropical Pacific Pelagic Ecosystem Size Structure: Observations and Theory
Source: PLoS One. 2013 Apr 19;8(4):e62341. doi: 10.1371/journal.pone.0062341 (PMC3631147; doi:10.1371/journal.pone.0062341)
Supplement: Table S1 — Mean species weight, length, and length-weight conversion factors. From left, columns indicate species, mean species weight as determined form length-weight conversions, mean length from those recorded by observers measuring every third fish from 2006–2011, a, b, and reference listing length-weight conversion factors. To convert length (L) in cm to weight (W) in g, the equation aLb = W was used. (DOCX) [file pone.0062341.s002.docx]

**Table S1. Mean species weight, length, and length-weight conversion factors.**

| **Species** | **Mean Weight (kg)** | **Mean Length (cm)** | ***a*** | ***b*** | **Reference*^a^*** |
| --- | --- | --- | --- | --- | --- |
| Blue Marlin (*Makaira nigricans*) | 224.0 | 166.3 | 0.0411 | 3.0363 | 1, 2 |
| Blue Shark (*Prionace glauca*) | 106.4 | 171.7 | 0.0025 | 3.3835 | 2 |
| Striped Marlin (*Tetrapturus audax*) | 93.5 | 132.8 | 0.0057 | 3.3760 | 2 |
| Shortbill Spearfish (*Tetrapturus angustirostris*) | 75.7 | 134.8 | 0.0005 | 3.8340 | 2 |
| Shortfin Mako Shark (*Isurus oxyrinchus*) | 48.3 | 177.3 | 0.0167 | 2.8470 | 2 |
| Swordfish (*Xiphias gladius*) | 42.0 | 105.5 | 0.0078 | 3.2100 | 2 |
| Yellowfin Tuna (*Thunnus albacares*) | 33.5 | 109.2 | 0.0129 | 3.1125 | 2, 3 |
| Opah (*Lampris guttatus*) | 30.2 | 101.6 | 0.0281 | 3.0000 | 2 |
| Bigeye thresher Shark (*Alopias superciliosus*) | 24.0 | 166.5 | 0.0238 | 2.6750 | 2 |
| Unidentified Tuna | 24.0 | 107.2 | 0.0155 | 3.0203 | -*^b^* |
| Bigeye Tuna (*Thunnus obesus*) | 22.5 | 113.7 | 0.0155 | 2.9725 | 2, 3 |
| Oceanic White-tip Shark (*Carcharinus longimanus*) | 19.0 | 141.8 | 0.0254 | 2.6910 | 2 |
| Albacore Tuna (*Thunnus alalunga*) | 17.1 | 104.7 | 0.0407 | 2.7800 | 2, 3 |
| Wahoo (*Acanthocybium solandri*) | 16.4 | 127.0 | 0.0024 | 3.2422 | 2, 4 |
| Escolar (*Lepidocybium flavobrunneum*) | 12.1 | 75.1 | 0.0353 | 2.9053 | 2, 5 |
| Mola (*Ranzania laevis* and *Mola mola*) | 8.8 | 51.4 | 0.0454 | 3.0500 | 2 |
| Skipjack Tuna (*Katsuwonus pelamis*) | 7.9 | 70.9 | 0.0085 | 3.2160 | 2 |
| Mahi Mahi (*Coryphaena hippurus*) | 7.4 | 87.3 | 0.0172 | 2.8868 | 2, 4 |
| Lancetfish (*Alepisaurus ferox*) | 7.1 | 107.7 | 0.0047 | 3.0000 | 2 |
| Great Barracuda (*Sphyraena jello*) | 5.9 | 99.9 | 0.0098 | 2.8750 | 2 |
| Pomfrets (*Taractichthys steindachneri* and *Brama japonica*) | 4.9 | 56.7 | 0.0160 | 3.0940 | 2 |
| Pelagic Stingray (*Pteroplatytrygon violacea*) | 3.0 | -*^c^* | - | - | 6 |
| Snake Mackerel (*Gempylus serpens*) | 0.8 | 101.7 | 0.0007 | 3.0000 | 2 |

From left, columns indicate species, mean species weight as determined from length-weight conversions, mean length from those recorded by observers measuring every third fish from 2006 – 2011, *a*, *b*, and reference listing length-weight conversion factors. To convert length (*L*) in cm to weight (*W*) in g, the equation *aL^b^ = W* was used.

*^a^*When multiple references are listed, the *a* and *b* values are an average of those listed in the literature.

*^b^*The *a* and *b* values for unidentified tuna are an average of those of all tuna listed in this table.

*^c^*Pelagic stingray lengths were not recorded.

1. Ortega-Garcia S, Klett-Traulsen A, Rodriguez-Sanchez R (2006) Some biological aspects of blue marlin (*Makaira nigricans*) in the recreational fishery at Cabo San Lucas, Baja California Sur, Mexico. Bulletin of Marine Science 79: 739-746.

2. Froese, R, Pauly D (eds.) (2012) FishBase. Available: http://www.fishbase.org. Accessed 7 June 2012.

3. Zhu G, Xu L, Zhou Y, Dai X (2008) Length-frequency compositions and weight-length relationships for bigeye tuna, yellowfin tuna, and albacore (Perciformes: scombrinae) in the Atlantic, Indian and Eastern Pacific Oceans. Acta Ichthyologica et Piscatoria 38:157-161.

4. Uchiyama JH, Boggs CH (2006) Length-weight relationships of dolphinfish, *Coryphaena hippurus*, and wahoo, *Acanthocybium solandri*: seasonal effects of spawning and possible migration in the central North Pacific. Marine Fisheries Review 68: 19-29.

5. Lorenzo JM, Pajuelo JG (1995) Biological parameters of the roudi escolar (*Promethicthys prometheus*) (Pisces: Gempylidae) off the Canary Islands. Fisheries Research 24: 65-71.

6. Pelagic stingray weight reference here
