# Supplementary material for: Fishery-Induced Changes in the Subtropical Pacific Pelagic Ecosystem Size Structure: Observations and Theory
Source: PLoS One. 2013 Apr 19;8(4):e62341. doi: 10.1371/journal.pone.0062341 (PMC3631147; doi:10.1371/journal.pone.0062341)
Supplement: Table S2 — Change in logbook catch rate estimate from statistically significant (P<0.01) linear regressions over 1996–2011, ordered by fish size. From left, columns indicate species, annual percent change in CPUE based on linear regression (P-values for significant trends in parentheses, insignificant fits denoted by a 0% change), and mean species weight as determined form length-weight conversion. (DOCX) [file pone.0062341.s003.docx]

**Table S2. Change in logbook catch rate estimated from statistically significant (*P*<0.01) linear regressions over 1996-2011, ordered by fish size.**

| **Species** | **% Annual Change in CPUE*^a^* (*P*-value)** | **Mean Weight in kg*^b^*** |
| --- | --- | --- |
| Blue Marlin (*Makaira nigricans*) | -4.2 (0.0002) | 224.0 |
| Blue Shark (*Prionace glauca*) | -3.4 (<0.0001) | 106.4 |
| Striped Marlin (*Tetrapturus audax*) | -5.0 (0.0007) | 93.5 |
| Shortbill Spearfish (*Tetrapturus angustirostris*) | -3.5 (0.007) | 75.7 |
| Shortfin Mako Shark (*Isurus oxyrinchus*) | 0 | 48.3 |
| Swordfish (*Xiphias gladius*) | 0 | 42.0 |
| Yellowfin Tuna (*Thunnus albacares*) | 0 | 33.5 |
| Opah (*Lampris guttatus*) | -4.2 (0.002) | 30.2 |
| Bigeye Thresher Shark (*Alopias superciliosus*) | 0 | 24.0 |
| Unidentified Tuna | -*^c^* | 24.0 |
| Bigeye Tuna (*Thunnus obesus*) | -2.3 (0.001) | 22.5 |
| Oceanic White-tip Shark (*Carcharinus longimanus*) | -*^c^* | 19.0 |
| Albacore Tuna (*Thunnus alalunga*) | -7.2 (<0.0001) | 17.1 |
| Wahoo (*Acanthocybium solandri*) | 0 | 16.4 |
| Escolar (*Lepidocybium flavobrunneum*) | 88.6 (<0.0001) | 12.1 |
| Mola (*Ranzania laevis* and *Mola mola*) | -*^c^* | 8.8 |
| Skipjack Tuna (*Katsuwonus pelamis*) | 0 | 7.9 |
| Mahi Mahi (*Coryphaena hippurus*) | 0 | 7.4 |
| Lancetfish (*Alepisaurus ferox*) | -*^c^* | 7.1 |
| Great Barracuda (*Sphyraena jello*) | -*^c^* | 5.9 |
| Pomfrets (*Taractichthys steindachneri* and *Brama japonica*) | 0 | 4.9 |
| Pelagic Stingray (*Pteroplatytrygon violacea*) | -*^c^* | 3.0 |
| Snake Mackerel (*Gempylus serpens*) | -*^c^* | 0.8 |

From left, columns indicate species, annual percent change in CPUE based on linear regression (*P*-values for significant trends in parentheses, insignificant fits denoted by a 0% change), and mean species weight as determined from length-weight conversion.

*^a^*from linear fit.

*^b^*as determined from observer recorded lengths and length-weight conversions.

*^c^*not included in logbook records.
